# Iris Recognition Using Image Moments and k-Means Algorithm

**DOI:** 10.1155/2014/723595

**Published:** 2014-04-01

**Authors:** Yaser Daanial Khan, Sher Afzal Khan, Farooq Ahmad, Saeed Islam

**Affiliations:** ^1^School of Science and Technology, University of Management and Technology, Lahore 54000, Pakistan; ^2^Department of Computer Science, AbdulWali Khan University, Mardan 23200, Pakistan; ^3^Faculty of Information Technology, University of Central Punjab, 1-Khayaban-e-Jinnah Road, Johar Town, Lahore 54000, Pakistan; ^4^Department of Mathematics, AbdulWali Khan University, Mardan 23200, Pakistan

## Abstract

This paper presents a biometric technique for identification of a person using the iris image. The iris is first segmented from the acquired image of an eye using an edge detection algorithm. The disk shaped area of the iris is transformed into a rectangular form. Described moments are extracted from the grayscale image which yields a feature vector containing scale, rotation, and translation invariant moments. Images are clustered using the k-means algorithm and centroids for each cluster are computed. An arbitrary image is assumed to belong to the cluster whose centroid is the nearest to the feature vector in terms of Euclidean distance computed. The described model exhibits an accuracy of 98.5%.

## 1. Introduction

Identification of individuals has been an important need over the ages. Conventionally identification documents like an identity card, passport, or driving license have been utilized for the purpose. Such identification methods have been evaded several times by use of forged documents. In the digital world a login and a password or a PIN code is used for identification. Besides shoulder surfing and sniffing several other techniques have evolved which are used to crack such codes and breach security. Undoubtedly a robust identification technique is essential for a safe and well supervised environment. This situation thrives the need of an identification technique using some biological inimitable features of a person. Numerous biological human features are peculiar and unique such as fingerprints, suture patterns, iris patterns, gait, and ear shapes. The patterns found in these structures are unique for every human; hence they can be used as an identification tool. In the recent past, use of iris image of a person for his identification has gained popularity. The radial and longitudinal muscles in the iris of an eye are responsible for the constrictions and dilation of the pupil. The pupil changes its size depending upon the light intensity the eye is exposed to. The muscles of iris form the texture of the iris while the presence or absence of a pigment forms the color of the iris. The color of the iris is genetically dependent, whereas the texture is not. The texture of iris forms random unique patterns for each human. A close observation of an iris may reveal pustules, rings, stripes, and undulations forming a unique pattern.

In the recent past, researchers have developed several mechanisms for matching the pattern that lies within the iris. In [1] the author employs a bank of Gabor filters to form a fixed length vector from the local and global iris characteristics. Iris matching is established based on the weighted Euclidean distance between the two iris images being compared. In another article by Monro et al., a technique is devised using discrete cosine transform. Iris coding is based on the differences of discrete cosine transform coefficients of overlapped angular patches from normalized iris images [2]. Certain researchers have employed various statistical models for the purpose. A nonparametric statistical model, namely, neural networks (NN), is used for pattern matching and data compression in [3–5]. The image processing technique using specially designed kernels for iris recognition is used to capture local characteristics so as to produce discriminating texture features in [6]. Several sorts of transformations also prove helpful in extracting useful features from an iris image. This feature vector is further used to form a classification approach for identifying a person based on his iris image [7, 8]. In the groundbreaking work by Daugman the iris recognition principle is based on the failure of statistical independence tests on iris phase structure encoded by multiscale quadrature wavelets. The combinatorial complexity of this phase information across different persons generates discriminating entropy enabling the most probable decision about a person's identity [9].

Most of the techniques based on feature extraction are designed for image of a certain fixed resolution. They fail to provide the desired result for the same images with different resolution. This characteristic implies that the model is not scale invariant. Techniques making use of NN incorporate a time taking training procedure. At times this training process may prove to be tricky rendering the model unable to yield quick results. On the other hand, some techniques that make use of certain filters may produce undesired results if the image is rotated which implies that such models are not rotation invariant. In this underlying paper a scale and rotation invariant technique for the same purpose is described. The proposed technique requires little training after which results are produced instantly. It is based on the use of image moments. Moments are properties that describe the characteristics of a certain distribution of data. Image moments (namely, Hu moments) are a quantitative measure of the shape of distribution formed by data collected as image pixel intensities and their locations [10].

In the proposed work the iris is segmented from an eye image. Image moments are computed from the segmented grayscale image. Classification of an iris is performed by the k-means algorithm. The composition of the paper is as described here.  Section 2 gives an overview of iris recognition process.  Section 3 explains the method used for iris segmentation.  Section 4 gives a method for transforming the radial information of the iris into a rectangular form.  Section 5 explains how this method can be further optimized. Image moments and method of computation of moments are described in Section 6.  Section 7 describes the adoption of k-means algorithm for clustering and classification using moments information. Some of the results are discussed in Sections 8 and 9 presents some conclusions.

## 2. Iris Recognition

Initially the image of an eye is acquired by a device called iriscope specifically designed for eye image acquisition at a high resolution. A large database of such images is collected having several classes. The iris within the image is segmented using an accurate and sufficiently fast technique. The iris image is of radial nature, rather than rectangular, which makes it unsuitable to be processed by any mathematical or statistical model of linear nature. There are two approaches to resolve this problem. The first approach is to adapt a model capable of processing data in its inherent radial form. Other approaches require transformation of the radial data into multidimensional linear form such that the information pertaining to iris texture is retained. In this piece of work the latter approach is adopted.

The information within the texture of the rectangular image may be used to form a probability density function. The image moments quantify the characteristics of this distribution. Using these raw moments translation, scale and rotation invariant moments are computed. Accumulated, these moments describe the characteristics of the pattern of the iris. This forms a feature vector which is later used for classification of iris images.

## 3. Iris Segmentation

**Figure 1:**
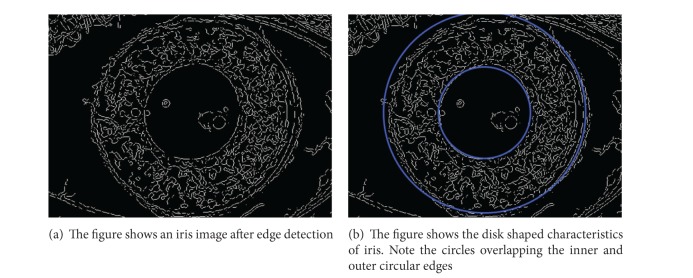
The figure depicts iris image after edge detection making disk shaped edges apparent.

**Figure 2:**
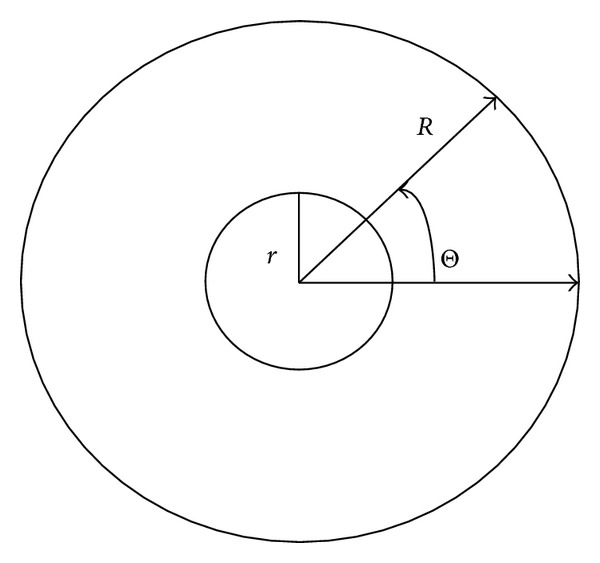
Transforming the radial iris into rectangular form.

Each image in the database contains the iris pattern which is of interest; the rest of the image is of no use and therefore is not processed. The iris is extracted from the image using the segmentation process described in [5]. The iris is modeled as a disk-like structure consisting of two concentric circles (see Figure 2). The noise in the eye image is suppressed using numerous iterations of median filtering [11]. The image with reduced noise is filtered to extract edges using an edge detection algorithm like the Canny [12] or the Sobel [13] filter as shown in Figure 1(a). Now using the resultant image the iris outline is extracted. The image is scanned top to bottom, left to right line by line. Each point on the outer and the inner edge is stored in two separate arrays. These points are further used to determine the center and the radii of the concentric circles forming the iris and the pupil as shown in Figure 1(b). Assuming that the outline of the iris is a circle, a point (*x*, *y*) on the circle with the center at (−*g*, −*f*) satisfies the equation

(1) $$\begin{array}{c} x^{2}+y^{2}+2gx+2fx+c=0. \end{array}$$

And the radius of the circle is given as

(2) $$\begin{array}{c} r=\sqrt{g^{2}+f^{2}-c}. \end{array}$$

Choosing any three arbitrary points from the array containing points of the circle, a system of simultaneous equations is formed. The solution to *c*, *f*, and *g* in terms of the selected three points are derived from the system and is given as

(3) g=(x12−x32+y12−y32)(y2−y1)   −(x12−x22+y12−y22)(y3−y1))  ×(2[(x3−x1)(y2−y1)    +(x1−x2)(y3−y1)])−1,f=x12−x22+y12−y22+2g(x1−x2)2(y2−y1),

where (*x*_1_, *y*_1_), (*x*_2_, *y*_2_), and (*x*_3_, *y*_3_) are the three arbitrary points.

Putting the values of *f* and *g* the value of *c* is determined from the following equation:

(4) $$\begin{array}{c} c=-x_{1}^{2}-y_{1}^{2}-2gx_{1}-2fy_{1}. \end{array}$$

Moreover the radius *r* is determined using (2). The center and the radii of both the concentric circles are determined in the described manner. The information within the inner circle is left out as it encompasses the pupil while the information bound in between the inner and outer circle contains the significant and unique iris pattern. Several triplets of circle points are used to compute the center of each circle. The best estimation of center is achieved by discarding extreme center points and then taking the mean of the rest of the points. For each center point (*x*_*i*_, *y*_*i*_) of inner circle and for each center point (*u*_*i*_, *v*_*i*_) for outer circle the mean (*x*_*c*_, *y*_*c*_) is computed as

(5) $$\begin{array}{c} x_{c}=\frac{\sum_{i=1}^{n}x_{i}+\sum_{i=1}^{n}u_{i}}{2n},\\ y_{c}=\frac{\sum_{i=1}^{n}y_{i}+\sum_{i=1}^{n}v_{i}}{2n}. \end{array}$$

Similarly, averages for the radius of the inner circle *r*_*m*_ and the radius for outer circle *R*_*m*_ are computed as

(6) $$\begin{array}{c} r_{m}=\frac{\sum_{i=1}^{n}r_{i}}{n},\\ R_{m}=\frac{\sum_{i=1}^{n}R_{i}}{n}. \end{array}$$

The pattern formed by the radial and longitudinal muscles of the iris is of interest. Further, this pattern is extracted to form a rectangular image. A computationally moderate solution to the problem must provide a faster transformation method. One such method is discussed in [5] which transforms the radial image into rectangular form and further makes use of the midpoint algorithm to optimize it as described in the next section.

## 4. Radial to Linear Transformation

An arbitrary Cartesian point (*x*′, *y*′) anywhere on the disk-like structure having parametric coordinates (*r*, *θ*) is given as

(7) $$\begin{array}{c} x^{\prime }=r\cdot \cos\left(\theta \right),\\ y^{\prime }=r\cdot \sin\left(\theta \right). \end{array}$$

**Figure 3:**
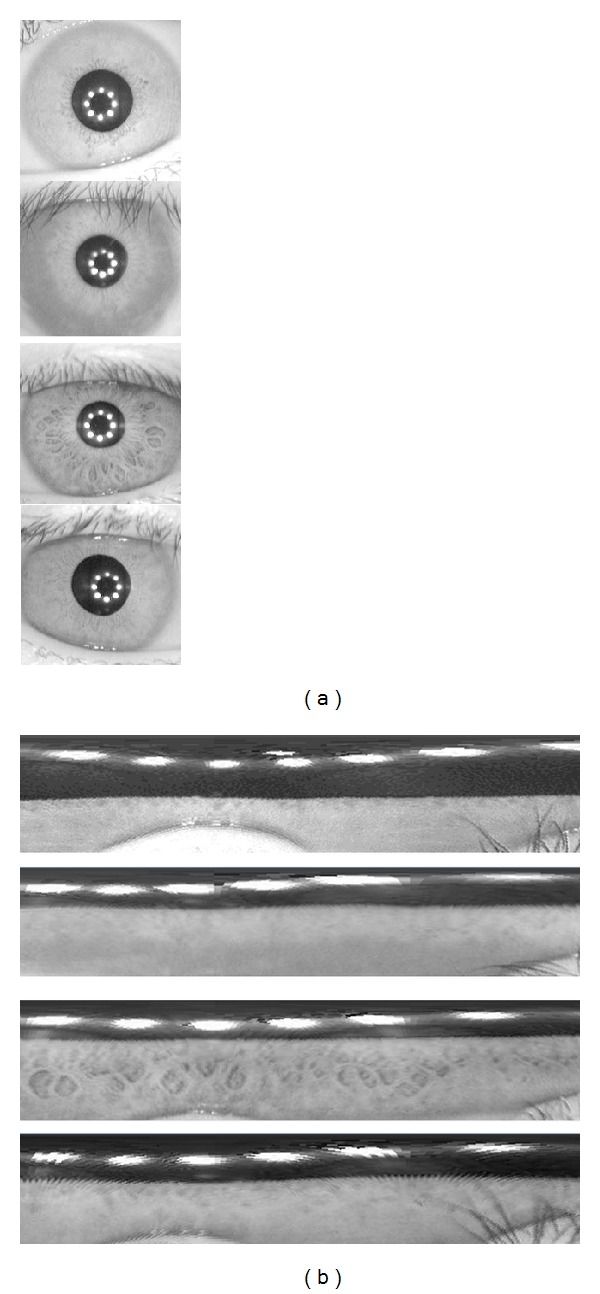
The figure illustrates iris images before and after radial to rectangular transformation.

Cartesian points along the line starting at parametric point (*r*_*m*_, *θ*) and ending at (*R*_*m*_, *θ*) are discretized at appropriate intervals and are placed in a column of a two-dimensional array. A number of columns are collected starting from *θ* = 0° and incrementing it in small steps up to *θ* = 360°. The collection of these columns forms a rectangular canvas containing the required iris pattern, (see Figure 3).

The computations required to compute each point within the disk shaped structures are reduced by exploiting the symmetric properties of a circle. A circular shape exhibits eight-way symmetry [14]. This means for any computed point (*a*, *b*) seven more points are determined that lie on the same circle using its symmetric properties. These seven points are described as (−*a*, *b*), (−*b*, *a*), (*a*, −*b*), (*b*, −*a*), (−*a*, −*b*), and (−*b*, −*a*) given that the center lies at the origin. In case the center lies at an arbitrary point, then these points are translated accordingly. Use of this symmetric property reduces the computations eightfold. Each point on the line is determined by incrementing the *x*-coordinate in discrete steps and calculating the corresponding value of *y*-coordinate using the line equation.

The *x*-coordinate and the *y*-coordinate of the pixels along a single line making an arbitrary angle *θ* can be determined incrementally while the starting pixel coordinates are (*x*′, *y*′) and the coordinates of the endpoint pixel are (*x*, *y*). Based upon the value of *x*-coordinate of previous pixel, the value of *x*-coordinate for next pixel is calculated by incrementing the previous value of *x*. This value of *x*-coordinate, say *x*_*m*_, is put into the following equation:

(8) $$\begin{array}{c} y_{m}=\frac{y-y^{\prime }}{x-x^{\prime }}\left(x_{m}-x^{\prime }\right)+y, \end{array}$$

which yields the corresponding *y*-coordinate.

## 5. Optimizing the Algorithm

The determination of points along a line is further optimized by the use of the midpoint method [14]. The computations required to yield a point along a line are reduced to mere addition of a small incremental value. The gradient *m* is computed for this purpose as given in the following equation:

(9) $$\begin{array}{c} m=\frac{y-y^{\prime }}{x-x^{\prime }}. \end{array}$$

Let

(10) $$\begin{array}{c} dy=y-y^{\prime },\\ dx=x-x^{\prime }, \end{array}$$

where the line end points are (*x*′, *y*′) and (*x*, *y*). In accordance with the midpoint method for straight line with tangent between 0 and 1 the value of Δ*E* = 2(*dy* − *dx*) and Δ*NE* = 2*dy*. Initially the control variable *d* = 2*dy* − *dx*. If the value of *d* is positive then East Pixel is chosen and if it is negative then North East Pixel is chosen. At each step *d* is updated by adding Δ*E* or Δ*NE* accordingly [5, 14].

## 6. Pattern Recognition Using Image Moments

A perceptive action performed on intricate structures needs to quantify its attributes. The state of any structure is quantifiable into data. Diversification of this data represents interaction or changes in the state. All such quantification methods generate finite data. Data by itself is insignificant, but the information implanted within the data is useful. Information is either extracted directly from the data itself or from the patterns formed by the arrangement of data. Researchers have devised various models for extracting information from data embedded in an image. Applications based on such models do not add to the contents of data rather they find hidden data patterns in order to extract interesting and useful information. A probability density can be formed for any data set. The parameters of the probability density function inform us about the general manner in which data is distributed. Moments are the characteristics of the probability density function which are based on the kurtosis and skewedness of the probability density function. Image moments describe the properties of a distribution formed using the pixel data of the image along its axes. The moments are typically chosen to depict a certain interesting property of the image. Such moment proves beneficial in extracting and summarizing the properties of the image in order to produce useful results. Properties of an image such as centroid, area, and orientation are quantified by this process. Another dividend of image moments is that they bring together the local and global geometric details of a grayscale image [15].

### 6.1. Extracting Moments from an Image

An image in the real world is modeled using a Cartesian distribution function *f*(*x*, *y*) in its analog form. This function is used to provide moments of the order of (*p* + *q*) over the image plane P and is generalized as

(11) Mpq=∫∫Pψpq(x,y)·f(x,y)dx dy; p,q=0,1,2,…,∞,

where *ψ*_*pq*_ is the basis function and *Ρ* is the image plane. Equation (11) yields a weighted average over the plane P. The basis function is designed such that it represents some invariant features of the image. Furthermore the properties of the basis function are passed onto moments. An image is of discrete nature; thus it is divided into pixels each having a discrete intensity level. Equation (11) is adopted for the digital image as

(12) $$\begin{array}{c} M_{pq}=\sum_{x}\sum_{y}\psi_{pq}\left(x,y\right)\cdot I\left(x,y\right);\;p,q=0,1,2,\ldots ,\infty , \end{array}$$

where *I*(*x*, *y*) is the intensity of a pixel in the digital image at the *x*th row and *y*th column.

In [10, 16] the authors prove that the two-dimensional continuous (*p* + *q*)th order moments are defined using the integral

(13) $$\begin{array}{c} M_{pq}=\iint_{-\infty }^{\infty }x^{p}y^{q}I\left(x,y\right)dx\;dy, \end{array}$$

where *f*(*x*, *y*) lies within some finite region of the *xy* plane. In case of digital image the integrals are replaced by summations, which is formulated as

(14) $$\begin{array}{c} M_{pq}=\overset{K}{\underset{x=1}{\sum }}\overset{L}{\underset{y=1}{\sum }}x^{p}y^{q}I\left(x,y\right), \end{array}$$

where *x* ^*p*^ *y* ^*q*^ is the basis function, *K* and *L* are the dimensions of the image, and the *M*_*pq*_ is the cartesian moment for the two-dimensional image. Note that this basis function is highly correlated, that is, nonorthogonal. The moment *M*_00_ represents the image, whereas the first order moments are used to find the center of the mass or the centroid of the image is given as

(15) $$\begin{array}{c} \overset{-}{x}=\frac{M_{10}}{M_{00}},\\ \overset{-}{y}=\frac{M_{01}}{M_{00}}, \end{array}$$

where $\left(\overset{-}{x},\overset{-}{y}\right)$ is the centroid.

### 6.2. Centralized Moments

Once the centroid is determined, it is used to compute the centralized moments. In [15] the central moments for two-dimensional data are given as

(16) $$\begin{array}{c} \mu_{pq}=\sum_{x}\sum_{y}{\left(x-\overset{-}{x}\right)}^{p}{\left(y-\overset{-}{y}\right)}^{q}I\left(x,y\right), \end{array}$$

where *μ*_*pq*_ are the central moments. Note that these are similar to Cartesian moments translated to the centroid. This depicts the translation invariant property of the centralized moments which are always akin to the centroid of the segmented object. Further simplification of (16) up to order 3 generates the following moments:

(17) μ00=M00,μ01=0,μ10=0,μ11=M11−x−M01=M11−y−M10,μ20=M20−x−M10,μ02=M02−y−M01,μ21=M21−2x−M11−y−M20+2x−2M01,μ12=M12−2y−M11−x−M02+2y−2M10,μ30=M30−3x−M20+2x−2M10,μ03=M03−3y−M02+2y−2M01.

### 6.3. Scale Invariant Moments

These moments are further made scale invariant as explained in [4, 5] and are given as

(18) $$\begin{array}{c} \eta_{pq}=\frac{\mu_{pq}}{\mu_{00}^{\gamma }}, \end{array}$$

where *η*_*pq*_ are scale normalized central moments and *γ* = (*p* + *q*)/2 + 1 where (*p* + *q*) ≥ 2.

### 6.4. Image Orientation

The second order central moments contain information about the orientation of the image. Using these moments a covariance matrix is derived. Let

(19) $$\begin{array}{c} \mu_{20}^{\prime }=\frac{\mu_{20}}{\mu_{00}}=\frac{M_{20}}{M_{00}}-{\overset{-}{x}}^{2},\\ \mu_{02}^{\prime }=\frac{\mu_{02}}{\mu_{00}}=\frac{M_{02}}{M_{00}}-{\overset{-}{y}}^{2},\\ \mu_{11}^{\prime }=\frac{\mu_{11}}{\mu_{00}}=\frac{M_{11}}{M_{00}}-\overset{-}{x}\overset{-}{y}, \end{array}$$

and then the covariance matrix is given as

(20) $$\begin{array}{c} \mathrm{cov}\left[I\left(x,y\right)\right]=\left(\begin{array}{cc} \mu_{20}^{\prime } & \mu_{11}^{\prime }\\ \mu_{11}^{\prime } & \mu_{02}^{\prime } \end{array}\right). \end{array}$$

The major and minor axes of the image intensity correlate with the eigenvectors of the given covariance matrix. The orientation of the image is described by the eigenvector of the highest eigenvalue. In [4] it is shown that the angle Θ is computed by the following equation:

(21) $$\begin{array}{c} \Theta =\frac{1}{2}\text{ta}{{\text{n}}^{-1}}_{}\left(\frac{2\mu_{11}^{\prime }}{\mu_{20}^{\prime }-\mu_{02}^{\prime }}\right), \end{array}$$

where

(22) $$\begin{array}{c} \mu^{\prime }\neq 0. \end{array}$$

Using (20) the eigenvalues of the covariance matrix are easily obtained and are given as

(23) $$\begin{array}{c} \lambda_{i}=\frac{\mu_{20}^{\prime }+\mu_{02}^{\prime }}{2}\pm \frac{\sqrt{4\mu_{11}^{\prime 2}+{\left(\mu_{20}^{\prime }-\mu_{02}^{\prime }\right)}^{2}}}{2}. \end{array}$$

Notice that these values are proportional to the square of the length of the eigenvector axes. The difference between the eigenvalues marks yet another important characteristic. It shows how elongated the image is. This property is termed eccentricity and is computed as

(24) $$\begin{array}{c} \sqrt{1-\frac{\lambda_{2}}{\lambda_{1}}}. \end{array}$$

### 6.5. Rotation Invariant Moments

Previously we have discussed translation and scale invariant moments. In [16] rotation invariant moments are derived which are usually termed as a Hu set of invariant moments. These are given as follows:

(25) I1=η20+η02,I2=(η20−η02)2+(2η11)2,I3=(η30−3η12)2+(3η21−η03)2,I4=(η30+η12)2+(η21+η03)2,I5=(η30−3η12)(η30+η12)  ×[(η30+η12)2−3(η21+η03)2]  +(3η21−η03)(η21+η03)  ×[3(η30+η12)2−(η21+η03)2],I6=(η20−η02)  ×[(η30+η12)2−(η21+η03)2]  +4η11(η30+η12)(η21+η03),I7=(3η21−η03)(η30+η12)  ×[(η30+η12)2−3(η21+η03)2]  −(η30−3η12)(η21+η03)  ×[3(η30+η12)2−(η21+η03)2],I8=η11[(η30+η12)2−(η03+η21)2]  −(η20−η02)(η30+η12)(η03+η21).

Every one of the rotation invariant moments extracts a characteristic attribute of the image. For example *I*_1_ represents the moment of inertia along the centroid while *I*_7_ extracts skew invariant properties which are useful in differentiating between images which are mirror reflections of each other [10, 15–17].

## 7. Clustering for Classification

By now the iris image has been segmented and transformed into a rectangular canvas. All described moments are applied and a feature vector is extracted, namely, $\bar{I}$. This vector contains translation, scale, and rotation invariant and orientation related moments. This vector corresponds to various features of the image; hence it is used for classification. An unsupervised approach is adopted for classification using k-means clustering algorithm. Using a set of multidimensional observations (*x*_1_, *x*_2_,…, *x*_*n*_), the k-means algorithm partitions the observations into *K* sets such that *K* ≤ *n* generating the set *S* = *S*_1_, *S*_2_,…, *S*_*k*_ so as to minimize the following objective function:

(26) $$\begin{array}{c} \underset{s}{\arg}\min\overset{K}{\underset{i=1}{\sum }}\sum_{x_{j}\in S_{i}}{\left|x_{j}-\mu_{i}\right|}^{2}, \end{array}$$

where *μ*_*i*_ is mean of all the observations in *S*_*i*_. Initially extracted moment vectors for an iris image sample are considered to be the initial mean.

The k-means Algorithm has two major steps, namely, the assignment step and the update step. The mean is used to assign each observation to a cluster in the assignment step. An observation is assigned a cluster whose mean makes the closest match. Formally this step generated the set *S*_*i*_ such that

(27) $$\begin{array}{c} S_{i}=x_{r}:\left|x_{r}-m_{i}\right|\leq \left|x_{r}-m_{j}\right|\;\forall 1\leq j\leq K. \end{array}$$

Also an observation *x*_*r*_ should be associated with exactly one *S*_*i*_ even if two or more differences are found comparable. The next step is based on the identification of a cluster for the observation established in the previous step. The mean for the cluster is recalculated as the centroid of the observations as given in following equation:

(28) $$\begin{array}{c} m_{i}=\frac{1}{\left|S_{i}\right|}\sum_{x_{j}\in S_{i}}x_{j}. \end{array}$$

Both steps are iterated and the centroid is readjusted. This process continues until there is no appreciable change in the means. At this stage the means have converged and no further training is required [18, 19].

## 8. Results

**Figure 4:**
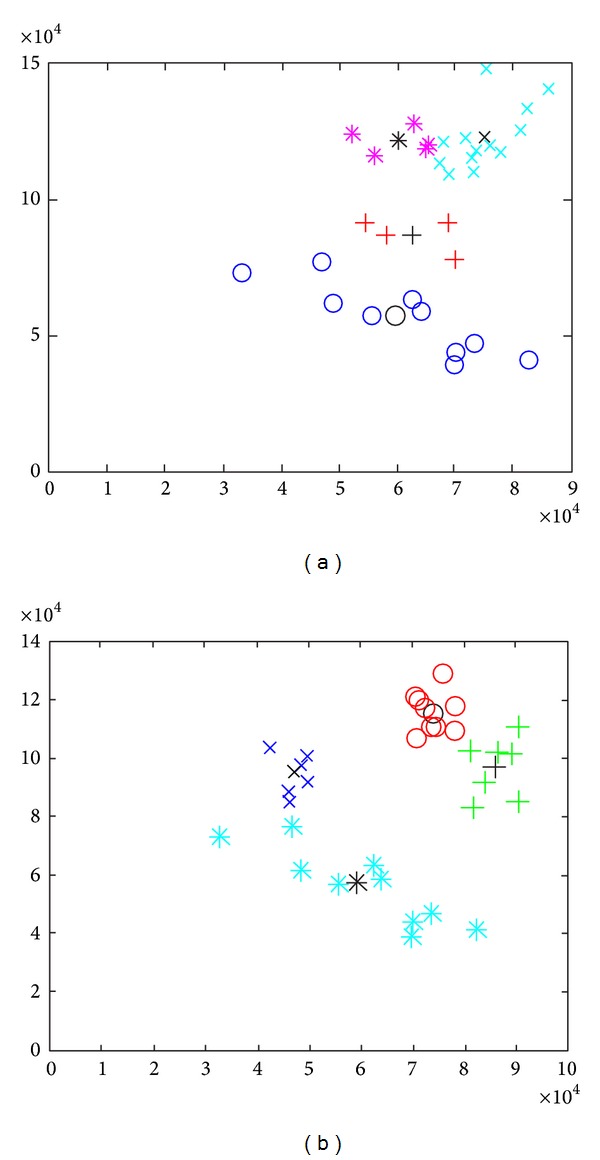
Each of (a) and (b) shows different clusters. Notice that all the clusters are linearly separable and can be distinguished by their Euclidean distance from the Centroid.

**Figure 5:**
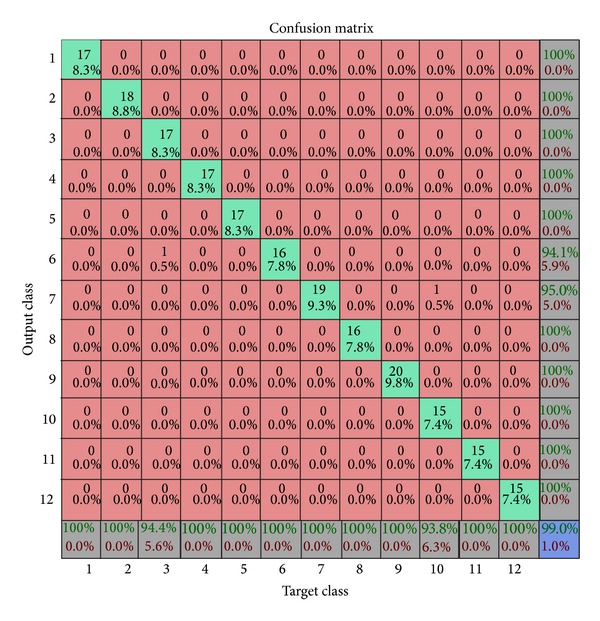
The figure shows the confusion matrix for some arbitrary classes, while the accuracy of the model for these classes is 99.0%.

The CASIA database containing thousands of images belonging to hundreds of different people is used to gather test results. Nearly one-fourth of the iris images from each class are retained as test case while the rest are used for training. The distorted images within the database are rejected. Iris portion of the image is marked out using the segmentation algorithm and is later transformed into a rectangular canvas. Further the grey scale rectangular canvas of iris is used to compute image moment vector. This vector contains information which is translation, scale, and rotation invariant and provides orientation information. Using the k-means algorithm each image is assigned to a cluster. The k-means algorithm is iterated until convergence is achieved and the centroid of each cluster is determined. Once the system is fully trained it is ready to accept an arbitrary input and provide a match. The model responds with the correlation of an arbitrary image moments vector to a cluster, if the image belongs to a known class. In Figure 4 various clusters formed are depicted; it also shows how the class of a sample is distinguished based upon the Euclidean distance of the feature vector of the sample from the centroid of an arbitrary cluster. Moreover Figure 5 shows a confusion matrix depicting the accuracy of the model. The confusion matrix shows that the accuracy of the model for certain arbitrary classes is 99.0% while the overall accuracy of the model for all the images in the database is estimated to be 98.5%. Moreover it also reports the level of confidence of match based on Euclidean distance of the sample from the centroid of the identified cluster. Level 0 is the highest which means that the Euclidean distance of the sample from the centroid of the cluster is low and level 4 is the lowest which indicates that the Euclidean distance of the sample from the centroid of any cluster does not lie within a stipulated threshold to confidently indicate a match. Figure 4 shows the clusters formed using the k-means algorithm.

**Figure 6:**
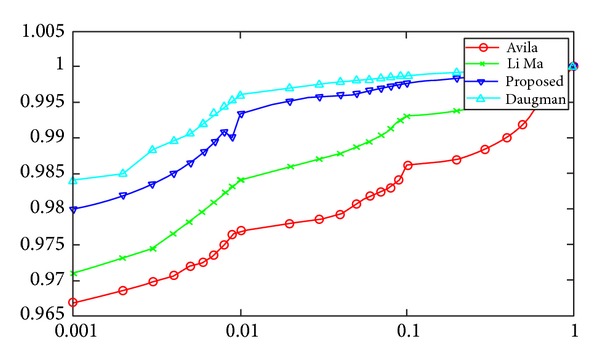
The figure illustrates the receiver operating characteristics distributions for different competitive techniques including the proposed one.

Furthermore a number of experiments were carried out to determine the accuracy and efficiency of the proposed model in comparison with other competitive models. In [20] the authors present a technique which extracts the features of iris using fine-to-coarse approximation at different resolution levels determined through discrete dyadic wavelet transform zero crossing representation. The resultant one-dimensional feature vector is used to find a match by computing various distances with arbitrary feature vectors. Ma et al. present yet another iris recognition technique using Gabor filters [1, 6]. The authors use a bank of Gabor filters to extract a fixed length feature vector signifying the global and local characteristics of the iris image. A match is established by computing the weighted Euclidean distance between feature vectors of arbitrary iris images. Daugman in [9] relies on the morphogenetic randomness of texture in the trabecular meshwork of iris. A failure of statistical independence test on two coded patterns from same iris indicates a match. This method extracts the visible texture of iris from a real time video image. The image is later encoded into a compact sequence of multiscale quadrature 2D Gabor wavelet coefficients. The most significant 256 bytes form the iris code. An exclusive OR operation is performed to generate a decision. All the above-mentioned techniques including the proposed technique are executed in order to obtain results. The genuine acceptance rate (GAR) and false acceptance rate (FAR) are observed for each technique. A receiver operating characteristics (ROC) distribution is plotted for each technique based on the results as shown in Figure 6. The ROC distribution comparatively highlights the accuracy along with the frequency of occurrence of errors of the proposed and other current state-of-art models. The following section briefly provides some discussion about the proposed system along with an interpretation of the ROC distribution formed.

## 9. Conclusion

Through analysis of data obtained after moments extraction a number of conclusions are inferred. Images of a certain iris differing in orientation yielded varying eigenvalues and eccentricity. However, a change in orientation of an image barely affects the values of rotation invariant moments while raw and scale invariant moments are affected. Change in orientation of an image affects the Euclidean distance of the moment vectors from the centroid. Despite this there still remains a great probability of the image to be classified correctly because of coherence in scale invariant moments. Although the model exhibits scale and rotation invariant attributes but some impairment is offered by luminosity of the image. Two arbitrary images of the same objects yield comparable moments if the luminosity is the same but they may yield differing moments in case luminosity is altered. In the underlying research work it is assumed that the luminosity level will be the same for all the images as each image is obtained by an iriscope working in similar conditions. The model provides resilience towards variation of scale and rotation as compared to other techniques which requires coherence of phase and size. The model can be further improved by incorporation of a technique that will process each image to provide uniform luminosity. Furthermore, the ROC distribution obtained (shown in Figure 6) from all the test cases shows that the performance of proposed model is comparable with Daugman method, while it yields a better performance than the methods described in [1, 6, 20].
